# Adenosine Triphosphate-Encapsulated Liposomes with Plasmonic Nanoparticles for Surface Enhanced Raman Scattering-Based Immunoassays

**DOI:** 10.3390/s17071480

**Published:** 2017-06-23

**Authors:** Xuan-Hung Pham, Eunil Hahm, Tae Han Kim, Hyung-Mo Kim, Sang Hun Lee, Yoon-Sik Lee, Dae Hong Jeong, Bong-Hyun Jun

**Affiliations:** 1 Department of Bioscience and Biotechnology, Konkuk University, Seoul 143-701, Korea; phamricky@gmail.com (X.-H.P.); greenice@konkuk.ac.kr (E.H.); kth890304@naver.com (T.H.K.); hmkim0109@konkuk.ac.kr (H.-M.K.); 2 School of Chemical and Biological Engineering, Seoul National University, Seoul 151-742, Korea; shlee.ucb@gmail.com (S.H.L.); yslee@snu.ac.kr (Y.-S.L.); 3 Department of Chemistry Education, Seoul National University, Seoul 151-742, Korea; jeongdh@snu.ac.kr

**Keywords:** adenosine triphosphate encapsulated liposomes, plasmonic nanoparticles, gold-silver alloys, surface enhanced Raman scattering

## Abstract

In this study, we prepared adenosine triphosphate (ATP) encapsulated liposomes, and assessed their applicability for the surface enhanced Raman scattering (SERS)-based assays with gold-silver alloy (Au@Ag)-assembled silica nanoparticles (NPs; SiO_2_@Au@Ag). The liposomes were prepared by the thin film hydration method from a mixture of l-α-phosphatidylcholine, cholesterol, and PE-PEG2000 in chloroform; evaporating the solvent, followed by hydration of the resulting thin film with ATP in phosphate-buffered saline (PBS). Upon lysis of the liposome, the SERS intensity of the SiO_2_@Au@Ag NPs increased with the logarithm of number of ATP-encapsulated liposomes after lysis in the range of 8 × 10^6^ to 8 × 10^10^. The detection limit of liposome was calculated to be 1.3 × 10^−17^ mol. The successful application of ATP-encapsulated liposomes to SiO_2_@Au@Ag NPs based SERS analysis has opened a new avenue for Raman label chemical (RCL)-encapsulated liposome-enhanced SERS-based immunoassays.

## 1. Introduction

Immunoassays have become an important analytical technique for the early diagnosis of disease and monitoring the efficacy of treatment [1]. The immunoassays generate a measurable signal in response to biomarker binding. In sandwich immunoassays, the secondary antibody is usually labeled with specialized markers to determine the number of secondary antibody molecules, representing the amount of captured biomarker molecules. Various readout tools have been developed and implemented for the last several decades, including scintillation counting [2], fluorescence [3], chemiluminescence [4], and electrochemical signal [5,6], enzymes [7], and quantum dots [1,8]. Among these methods, radio-labeled immunoassays (RIAs) and enzyme-linked immunosorbent assays (ELISAs) are common because of their low cost and convenience [1]. Fluorescence-based techniques have been widely used as a diagnostic tool for immunoassays. However, they have several intrinsic drawbacks, including a poor limit-of-detection (LOD), photobleaching, and limit of multiplex detection [1].

Because of the strong enhancement of Raman signals for molecules adsorbed on metal nanostructures, such as silver and gold, surface enhanced Raman scattering (SERS) can overcome the inherent weaknesses of Raman spectroscopy [1]. In addition, the nondestructive and ultrasensitive, as well as its multiplex capability, allow the SERS technique to be effectively utilized in sensitive detection of analytes [9,10,11,12,13,14,15,16,17,18,19,20,21]. Therefore, SERS technique is promising in various applications, including analytical studies, clinical diagnoses, and biomolecule detection [22,23,24,25,26]. Because the structures and components of metallic nanoparticles (NPs) are critical factors affecting the sensitivity and reproducibility of the SERS signal, various shapes of Au and Ag NPs have been studied as sensitive SERS substrates. Recently, we developed a novel SERS substrate, SiO_2_@Au@Ag NPs, which exhibited a sensitive and reproducible SERS signals, wherein the “hot spot” could be controlled carefully by varying the concentration of Ag^+^ [27,28].

Liposomes have been widely used in drug delivery, gene transfection, cosmetic formulations, and medical imaging because of their biocompatibility, ease of surface functionalization, large surface area, large internal volume, and encapsulation capability [29,30,31,32,33]. Because they are composed of a phospholipid bilayer surrounding an aqueous core, liposomes can entrap hydrophilic molecules in the aqueous core or hydrophobic molecules in the lipid bilayer; these molecules include visible or fluorescent dyes [34], electrochemically active species [35], DNA [36], and enzymes [37]. Thus, liposomes have been utilized as an assay platform for enhancing signal amplification with fluorescence detection [38], flow injection analysis [39], high-throughput microtiter plates [40,41,42], and array-based assays [43] for analytes of environmental, clinical, food safety, and national security interest [33]. In particular, liposomes have been utilized in great success as an amplification system in immunoassays because they can carry various molecules and receptor ligands owing to their large surface area and internal volume. Indeed, liposomes have shown important applications in homogeneous and heterogeneous immunoassays by using antigen or antibody conjugated liposomes in flow-injection liposome immunoassays and liposome immunosensors [44]. In principle, the signals from liposomes can be detected by measuring intact molecules or their encapsulated molecules after rupture [45,46]. Assay methods involving liposome rupture have several advantages; e.g., prevention of static or collisional quenching of fluorescent molecules by the lipid bilayer, thereby resulting in higher sensitivity [45].

Recently, liposomes combined with Ag or Au NPs have been utilized as a SERS-based intracellular drug nanocarrier [47,48]. Liposomes were also utilized with gold nanosphere array substrate to enhance the SERS signals for sensitive immunoassays [23]. Because the method was combined with array substrate, additional transfer step to the SERS substrate is needed for immunoassay. Moreover, preparation of uniform sized gold nanosphere immobilized array as a SERS substrate is complicated, and can be contaminated by capping agent, such as CTAB, which can limit high-throughput analysis with low signal to noise. In this study, we demonstrate that adenosine triphosphate (ATP), as a Raman label chemical (RLC) encapsulated in liposomes, can be utilized in obtaining enhanced SERS signals during immunoassay by using Au@Ag alloy-assembled silica NPs (SiO_2_@Au@Ag NPs) as a SERS substrate. Our approach can allow us to avoid the complexity and contamination of preparation process of SERS substrate and expand the high throughput capability of the SERS-based immunoassays.

## 2. Experimental

### 2.1. Materials

Tetraethylorthosilicate, APTS, silver nitrate (AgNO_3_), THPC, gold (III) chloride trihydrate (HAuCl_4_), ascorbic acid, polyvinylpyrrolidone (MW: 40000), PBS tablets, chloroform, ATP, and Tween 20 were purchased from Sigma-Aldrich (St. Louis, MO, USA) and used without further purification. Phosphatidylcholine (PC), cholesterol, and phosphoethanolamine-conjugated biotin (PE-PEG2000-biotin) were acquired from Avanti Polar Lipids (Alabaster, AL, USA). Ethyl alcohol (EtOH), and aqueous ammonium hydroxide (NH_4_OH, 27%) were purchased from Daejung (Siheung, Korea).

### 2.2. Preparation of SiO_2_@Au@Ag NPs

SiO_2_@Au@Ag NPs were prepared as previously reported [27,28]. Au-Ag core-shell NPs were prepared in aqueous medium by reducing and depositing the Ag source with ascorbic acid on gold NPs in the presence of PVP. Briefly, 100 µg SiO_2_@Au NPs (100 µL) was dispersed in 0.7 mL PVP (1 mg/mL). Silver nitrate (10 mM, 100 µL) was added to the solution, followed by addition of 10 mM ascorbic acid (100 µL). This solution was incubated for 1 h to reduce Ag+ ions to Ag metal. The resulting SiO_2_@Au@Ag NPs were obtained by centrifugation at 8500 rpm for 15 min and washed several times with EtOH to remove excess reagent. The SiO_2_@Au@Ag NPs were redispersed in 1.0 mL absolute EtOH.

### 2.3. Incorporation of ATP into SiO_2_@Au@Ag NPs

ATP solution (1 mL, 10 mM in PBS) was added to SiO_2_@Au@Ag NPs (100 µg), and the suspension was stirred vigorously for 2 h at 25 °C. The colloids were centrifuged and washed several times with EtOH. The NPs were redispersed in 1.0 mL absolute EtOH.

### 2.4. Preparation of ATP-Encapsulated Liposomes

ATP-encapsulated liposomes were prepared from a mixture of PC, cholesterol, and PE-PEG2000 (molar ratio, 70:10:20) dissolved in chloroform by the thin film hydration method [49,50,51,52]. Briefly, PC (5 mg), cholesterol (0.3 mg), and PE-PEG2000 (5.7 mg) were dissolved in 1 mL chloroform. The solvent was evaporated to form a thin layer of PC under nitrogen flow and vacuum for 15 min to ensure complete evaporation. The obtained thin film of PC was hydrated with ATP solution in PBS (50 mM, 5 mL) to obtain liposomes at a final concentration of 1 mg/mL. Multilamellar liposomes were formed by mixing until the solution became cloudy, followed by sonication for 1 min at room temperature. The vesicular solution was then passed through a 100-nm polycarbonate filter using a mini-extruder (Avanti) to produce suspension of liposomes. The suspension of liposome was then dialyzed with distilled water to remove any unencapsulated ATP molecules using a dialysis cassette (G2, 3500 MWCO; Thermo Scientific Inc., Waltham, MA, USA) for 24 h. The final solution was stored at 4 °C until use. The final liposomes were stable for 2–3 weeks at 4 °C. The sizes of the liposomes were determined using dynamic light scattering (Nano ZS90 (ZE N3690), Malvern Instrument Ltd., Worcestershire, UK).

### 2.5. Lysis of ATP-Encapsulated Liposomes and Immobilization of ATP on the SiO_2_@Au@Ag NPs

ATP-encapsulated liposomes (60 µL) were mixed with SiO_2_@Au@Ag NPs (100 µg) and 100 µL PBS containing 0.1% Tween 20 (PBST). The mixture was incubated at room temperature for 1 h. The colloids were centrifuged at 13,000 rpm for 15 min and washed several times with ethanol to remove unbound ATP and lipids. The NPs were redispersed in 100 µL absolute EtOH.

### 2.6. SERS Measurement of the SiO_2_@Au@Ag NPs

SERS signals of our materials were measured using a confocal micro-Raman system (LabRam 300, JY-Horiba, Tokyo, Japan) equipped with an optical microscope (BX41, Olympus, Tokyo, Japan). The SERS signals were collected in a back-scattering geometry using ×10 objective lens (0.90 NA, Olympus) and a spectrometer equipped with a thermoelectric cooled CCD detector. A 532 nm diode-pumped solid-state laser (CL532-100-S; Crystalaser, Reno, NV, USA) was used as the photo-excitation source, with 10 mW laser power at the sample. The strong Rayleigh scattered light was rejected using a long-pass filter. All SERS spectra were integrated for 5 s. The spot size of the laser beam was about 2 µm.

## 3. Result and Discussion

### 3.1. Preparation of ATP-Encapsulated Liposomes and SiO_2_@Au@Ag NPs

**Scheme 1:**
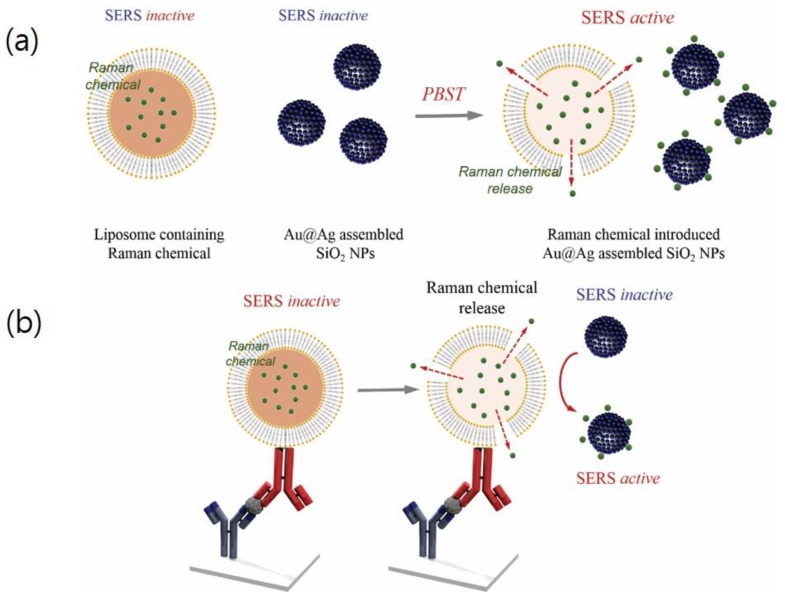
(**a**) Illustration of RLC-encapsulated liposome-enhanced SERS on Au@Ag-assembled silica NPs; (**b**) Suggested applications of RLC-encapsulated liposome-enhanced SERS-based immunoassays.

We designed and fabricated ATP-encapsulated liposomes that could release ATP only when the liposome structure was ruptured for SERS-based immunoassays as shown in Scheme 1. For this, ATP encapsulated liposomes and gold-silver alloy (Au@Ag)-assembled silica NPs (SiO_2_@Au@Ag) were prepared, separately. Both liposomes and SiO_2_@Au@Ag NPs alone were inactive for SERS measurement. However, when the liposome’s structure is broken, and the ATP is released, a strong SERS signal could be obtained, because the released ATPs are immobilized on SiO_2_@Au@Ag NPs.

As a Raman label chemical (RLC), ATP was selected. Although thiophenols and their derivatives, such as 4-aminothiophenol and 4-mercaptobenzoic acids, have been commonly used as a RLC because of their simple characteristic Raman bands, [22] the poor solubility of thiophenols in aqueous solution has limited their application in liposome preparation. Although Rhodamine 6G, Cy3, Cy5, crystal violet, and malachite green have also been used as RLCs for giving enhance SERS signals [23,53], their complicated SERS bands limit their use for analysis, especially for multiplexing [1].

Additionally, these RLCs are thought to be harmful to animals and the environment [1,54,55]. Therefore, we utilized ATP as a RLC because it is highly soluble in aqueous solution, and has simple, characteristic, and strong Raman bands. The ATP-encapsulated liposomes were prepared by using the thin film hydration method from a mixture of phosphatidylcholine (PC), cholesterol, and PE-PEG2000 in chloroform; evaporation of the solvent to form a thin film, followed by hydration with ATP solution (50 mM in PBS). The resulting vesicular solution was then passed through a 100-nm polycarbonate filter using a mini-extruder and dialyzed in distilled water to remove any unencapsulated ATP molecules to produce suspensions of liposomes.

Then, as a sensitive and reproducible SERS substrate, SiO_2_@Au@Ag NPs were prepared by Ag shell coating on Au NPs immobilized silica NPs, as recently reported. Briefly, colloidal Au NPs (2–3 nm) were prepared by reducing HAuCl_4_ with tetrakis(hydroxymethyl)phosphonium chloride (THPC), as reported by Duff et al. [56], with some modifications. Silica NPs were prepared by the Stober method, and the surface of the NPs was functionalized with amino groups using 3-aminopropyltriethoxysilane (APTS) [27,28]. The Au NPs were immobilized on the aminated silica NPs by gentle shaking. As shown in Figure S1, approximately 2300 Au NPs were assembled on the surfaces of the aminated silica NPs. The Ag shell was formed selectively on the surface of Au NPs on silica when AgNO_3_ was reduced in the presence of ascorbic acid and polyvinylpyrrolidone (PVP). The thickness of Ag NPs layer was controlled by adjusting the concentration of ascorbic acid, while the quantity of SiO_2_@Au NPs and the concentration of AgNO_3_ were fixed as 200 µg and 300 µM, respectively. The size of Au@Ag alloy was 45.7 ± 17.4 nm, which was measured by transmission electron microscopy (TEM) and analyzed by ImageJ software. The final size of SiO_2_@Au@Ag NPs was approximately 250 nm. The optical properties of SiO_2_@Au@Ag in the absence and presence of ATP was investigated in Figure S2. SiO_2_@Au@Ag showed the broad absorbance band in the range of 320 to 800 nm with a maximum peak at 457 nm [27], while ATP exhibited the characteristic peak of adenosine ring at 260 nm [57]. When ATP was added into the SiO_2_@Au@Ag solution, the maximum peak position showed a little shift and their intensities were also increased slightly. Therefore, we must use SERS to measure ATP in our study. To confirm that ATP was immobilized on the SiO_2_@Au@Ag, we further analyzed the materials by Raman spectroscopy.

### 3.2. Optimization of SERS Measurement of ATP in the Presence of SiO_2_@Au@Ag NPs

**Figure 1:**
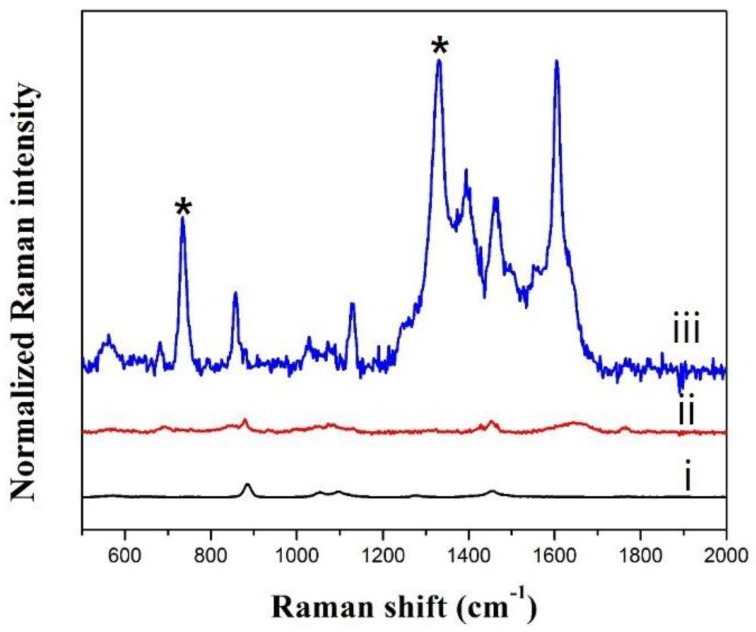
Surface enhanced Raman scattering (SERS) spectra of (**i**) SiO_2_@Au@Ag NPs, (**ii**) 10 mM ATP, and (**iii**) SiO_2_@Au@Ag NPs in the presence of 10 mM ATP. The concentrations of SiO_2_@Au@Ag NPs were 1 mg/mL in ethanol solution, respectively.

The SERS bands of ATP were investigated in the absence and the presence of SiO_2_@Au@ Ag NPs as a SERS substrate, and the results are shown in Figure 1. The SERS signal of SiO_2_@Au@Ag NPs in solid state observed at 563, 798 and 1090 cm^−1^ (Figure S3). However, the SERS of SiO_2_@Au@Ag NPs in ethanol solution appeared at 884, 1053, 1096, 1277, and 1454 cm^−1^ (spectrum i), which could be the SERS bands of ethanol. Similarly, ATP in ethanol solution showed the same SERS bands of ethanol (spectrum ii), because of low Raman signal of ATP without the metal surface. In contrast, ATP bands were clearly obtained with SiO_2_@Au@Ag NPs because of the electromagnetic enhancement by the NPs. Five characteristic Raman bands of adenosine were detected in the region from 500 to 2000 cm^−1^ (spectrum iii). The strong Raman band at 734 cm^−1^ was assigned to the in-plane breathing vibration of adenine. The peaks at 1142 and 1432 cm^−1^ were assigned to C-C stretching and C-N stretching vibrations, respectively [58]. The band located at 1335 cm^−1^ could be assigned to the bending vibration of C-H and the stretching vibrations of C-N, N-C-N, and C-C-N. The signal appearing at 1395 cm^−1^ was assigned to the bending vibrations of N-H and C-H. The band located at 1604 cm^−1^ corresponded to the ring breathing vibration of C=C [59,60,61,62]. The spectra shown in Figure S4 demonstrated that the intensities of the SERS signals of adenosine on the SiO_2_@Au@Ag substrate increased as the adenosine concentration increased in the range of 1 µM to 10 mM, particularly the band at 734 cm^−1^. Therefore, we chose the SERS band at 734 cm^−1^ for detection of ATP in subsequent analyses.

**Figure 2:**
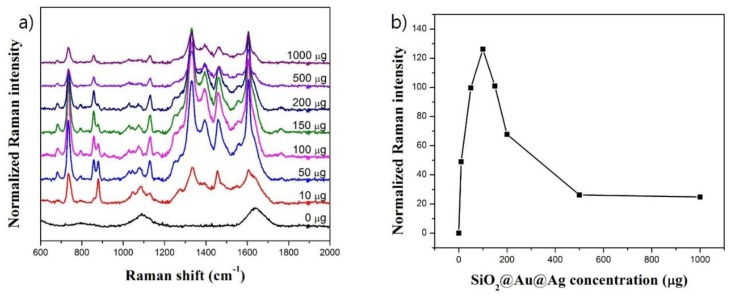
(**a**) SERS spectra and (**b**) plot the height of SERS peak at 734 cm^−1^ versus various concentration of SiO_2_@Au@Ag NPs. The concentration of SiO_2_@Au@Ag NPs ranged from 10 to 1000 μg and the concentration of ATP were 10 mM.

According to Ding et al., the mass and concentration of SERS materials may have significant effects on SERS performance [63]. Specifically, higher loading of silver increases the background signal of SERS, leading to decreased SERS signals. In this study, we performed SERS measurements of ATP (10 mM) with 10–1000 μg of SiO_2_@Au@Ag NPs. Figure 2 revealed that few SiO_2_@Au@Ag NPs could have been available as hotspots for SERS. Thus, a weak SERS signal of ATP was observed with 10 µg of the NPs. The SERS signals of ATP increased dramatically from 10 to 100 µg of SiO_2_@Au@Ag NPs, reaching a maximum value at 100 µg. However, the SERS signals of ATP with large amounts of SiO_2_@Au@Ag NPs (from 150 to 1000 µg) decreased dramatically because large number of NPs increased the background signal of the NPs and at the same time, decreased the number of ATP molecules adsorbed on the surface of the NPs. Therefore, the optimal amount of the NPs was set as 100 μg for further analysis.

### 3.3. Preparation of ATP-Encapsulated Liposomes

The ATP-encapsulated liposomes were prepared by the thin film hydration method. The solution of PC, cholesterol, and PE-PEG2000 in chloroform was evaporated to form a thin film, and hydrated with 50 mM ATP solution in PBS. The size of liposomes was ca. 140 ± 17 nm in diameter, which was measured by dynamic light scattering (Figure S5). Assuming that the lipid bilayer thickness is 5 nm and the average surface area of the head group per lipid (αL) for PC, phosphoethanolamine, and cholesterol is 0.65 ± 0.01, 0.52 ± 0.01, and 0.41 nm^2^, respectively [64], the αL value obtained for the liposomes is 0.6 nm^2^/lipid. Therefore, the number of lipid molecules can be calculated as 191,016 molecules/liposome. Given that the total concentration of lipids used to prepare the liposomes is 84.6 μM, the number of liposomes per milliliter can be calculated as 2.67 × 10^11^ [52]. According to Bui et al. [52], 50% of liposomes can be lost during extrusion and dialysis, the final concentration of liposome would be 1.33 × 10^11^ liposomes/mL after dialysis.

### 3.4. Effect of ATP-Encapsulated Liposomes on SERS Signal

**Figure 3:**
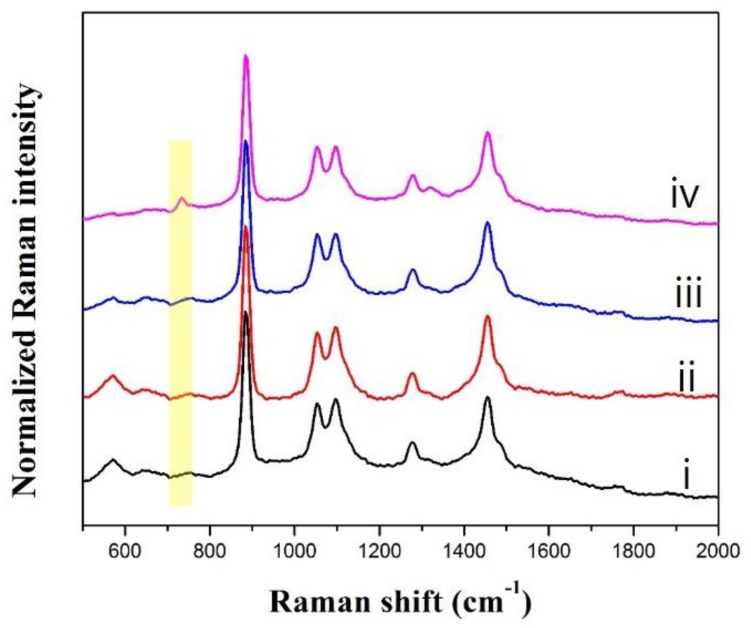
SERS spectra of (**i**) SiO_2_@Au@Ag, (**ii**) ATP encapsulated liposome, (**iii**) SiO_2_@Au@Ag in the presence of PBST and (**iv**) SiO_2_@Au@Ag in the presence of PBST and of ATP encapsulated liposome and PBST. The presence of ATP on the surface of SiO_2_@Au@Ag was confirmed at 734 cm^−1^.

To investigate the effects of ATP-encapsulated liposomes on the SERS signal, ATP-encapsulated liposomes were incubated with SiO_2_@Au@Ag NPs in the presence of PBS containing 0.1% Tween 20 (PBST). As shown in Figure 3, ATP-encapsulated liposomes did not exhibit any SERS bands because of the low Raman signal of ATP without the metal surface (Figure 3ii). The SERS spectra of SiO2@Au@Ag NPs in the absence (Figure 3i) and presence of PBST (Figure 3iii) were the same, indicating that PBST did not affect the SERS spectrum of SiO_2_@Au@Ag NPs. However, when SiO_2_@Au@Ag NPs were incubated with PBST and ATP-encapsulated liposomes, the SERS band at 734 cm^−1^ was clearly observed because the Tween 20 broke down the structure of the liposomes and ATP was released into the solution. Subsequently, ATP was absorbed on the surface of SiO_2_@Au@Ag NPs, and subsequently, SERS bands appeared, as observed in Figure 3iv.

**Figure 4:**
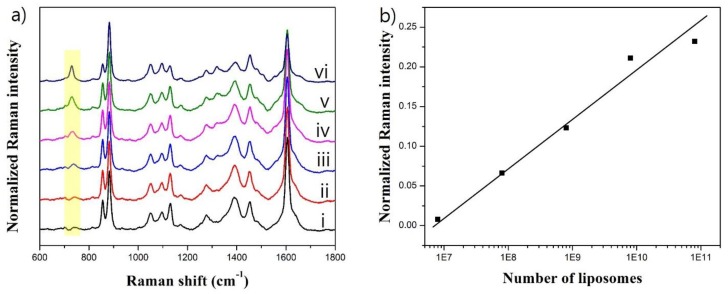
(**a**) SERS spectra and (**b**) calibration plot at 734 cm^−1^ of SiO_2_@Au@Ag NPs in the presence of various concentrations of liposomes. The numbers of liposomes were (i) 0, (ii) 8 × 10^6^, (iii) 8 × 10^7^, (iv) 8 × 10^8^, (v) 8 × 10^9^, and (vi) 8 × 10^10^. The concentration of SiO_2_@Au@Ag NPs was fixed at 100 μg. The characteristic peak of ATP was at 734 cm^−1^.

Finally, the effects of the number of ATP-encapsulated liposome on the SERS signal of SiO_2_@Au@Ag NPs were also assessed. The intensity of the SERS band at 734 cm^−1^ decreased as the number of liposomes decreased (Figure 4).

The SERS signal at the 734 cm^−1^ band almost disappeared when the number of ATP-encapsulated liposomes dropped to 8 × 10^6^. In addition, the SERS intensity of SiO_2_@Au@Ag NPs increased to the logarithm of number of ATP-encapsulated liposomes in the range of 8 × 10^6^ to 8 × 10^10^, with the square of the coefficient of multiple correlation (R2) of 99%. This result indicated that the detection limit of liposome was calculated to be 1.3 × 10^−17^ mol. (see Supplementary Materials). The successful application of ATP-encapsulated liposome lysis with SiO_2_@Au@Ag NPs to the SERS detection has opened a novel approach for Raman chemical-encapsulated liposome-enhanced SERS-based immunoassays, as illustrated in Figure S6. In this approach, ATP as a RLC was encapsulated in liposomes, and the surface was conjugated with detection antibodies. The concentration of antigen could be detected by analyzing the quantity of the Raman chemical-encapsulated liposome-conjugated detection antibody, which was determined by SERS measurement in the presence of plasmonic NPs after liposome lysis.

## 4. Conclusions

Here, we demonstrate that RLC-encapsulated liposome can be applied in plasmonic SERS measurement. ATP as a RLC was encapsulated using the thin film hydration method. The conditions for SERS measurement of ATP were optimized in the presence of SiO_2_@Au@Ag NPs. As a result, the SERS intensity of ATP increased with the logarithm of number of ATP-encapsulated liposomes after lysis in the range of 8 × 10^6^ to 8 × 10^10^. The detection limit of liposome was calculated to be 1.3 × 10^−17^ mol. Thus, ATP-encapsulated liposomes were successfully applied to the detection of RLC with SiO_2_@Au@Ag NPs, establishing a new analytical field for RLC-encapsulated liposome-enhanced SERS-based immunoassays.

## Supplementary Materials

The Supplementary Materials are available online at http://www.mdpi.com/1424-8220/17/7/1480/s1.
