# Supplementary material for: Adenosine Triphosphate-Encapsulated Liposomes with Plasmonic Nanoparticles for Surface Enhanced Raman Scattering-Based Immunoassays
Source: Sensors (Basel). 2017 Jun 23;17(7):1480. doi: 10.3390/s17071480 (PMC5539552; doi:10.3390/s17071480)
Supplement: Supplementary file 1 [file sensors-17-01480-s001.pdf]

Adenosine Triphosphate-Encapsulated Liposomes with Plasmonic Nanoparticles for Surface Enhanced Raman Scattering-Based Immunoassays

Xuan-Hung Pham, Eunil Hahm, Tae Han Kim, Hyung-Mo Kim, Sang Hun Lee, Yoon-Sik Lee, Dae Hong Jeong and Bong-Hyun Jun *

The detection limit of liposome number in our system was 8 × 10^6 units. When we change the liposome unit number to mol, it is equal to 1.3 × 10^{-17} mol: (8 × 10^6 units) × (1 mol/6.023 × 10^{23} units) = 1.3 × 10^{-17} mol.

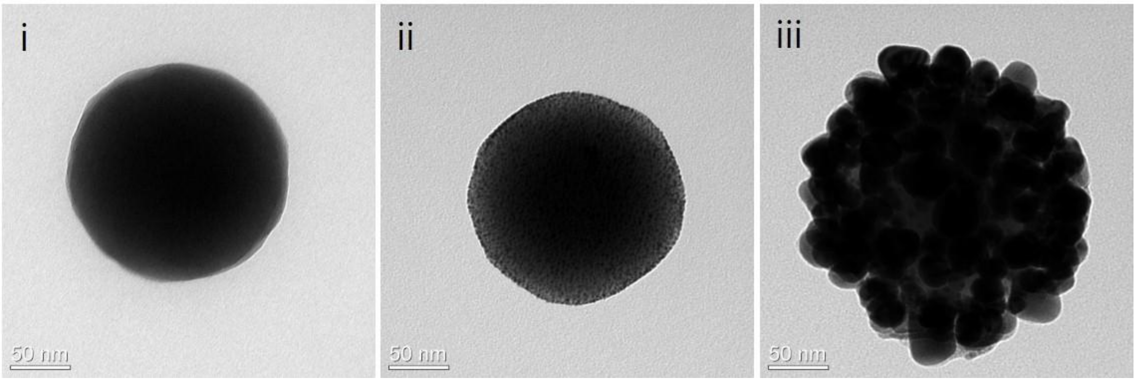

Figure S1. TEM images of (i) SiO_2, (ii) SiO_2@Au and SiO_2@Au@Ag nanoparticles synthesized at 200 µg SiO_2 and 300 µM $AgNO_3$.

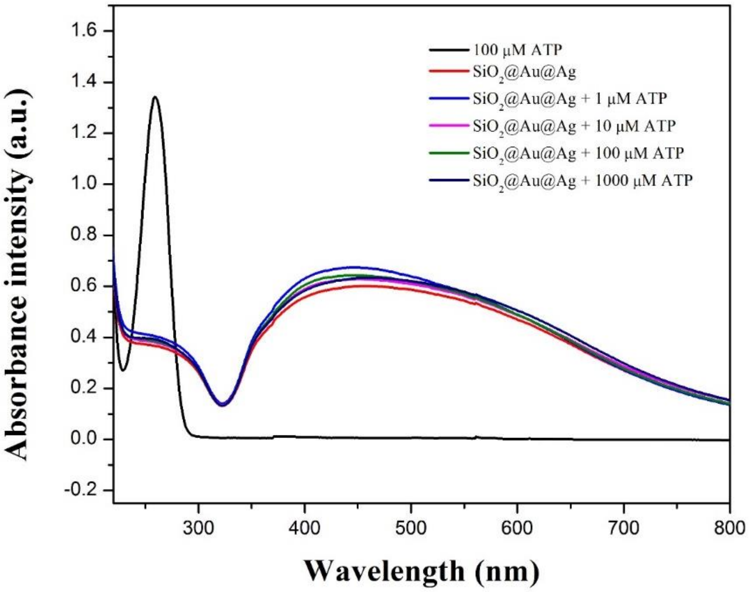

Figure S2. UV-Vis spectra of 100 µM adenosine triphosphate (ATP), SiO_2@Au@Ag in the presence of ATP in the range of 0 to 1000 µM. SiO_2@Au@Ag concentration is 10 µg/mL.

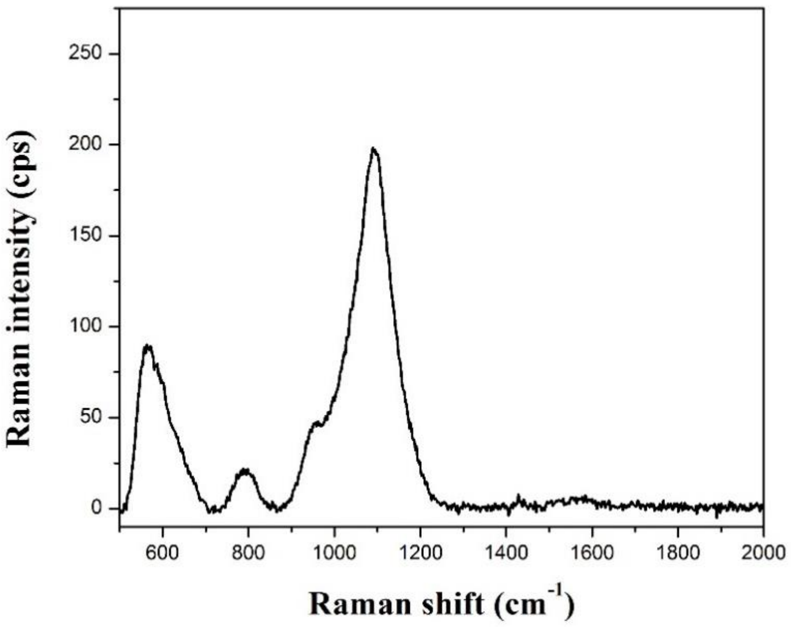

Figure S3. Raman spectrum of SiO_2@Au@Ag in solid state.

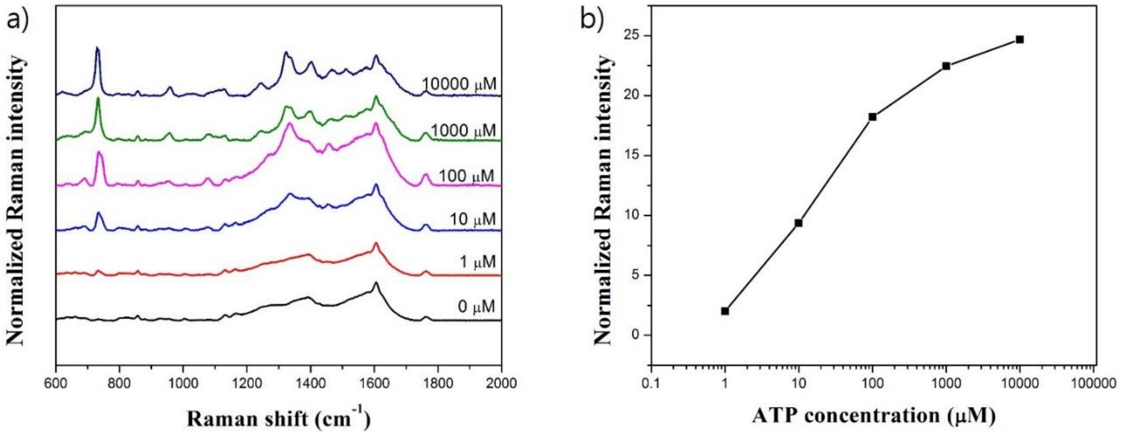

Figure S4. (**a**) Raman intensity and (**b**) calibration plot of SiO_2@Au@Ag in the presence of various concentration of adenosine triphosphate. SiO_2@Au@Ag concentration is 1 mg/mL.

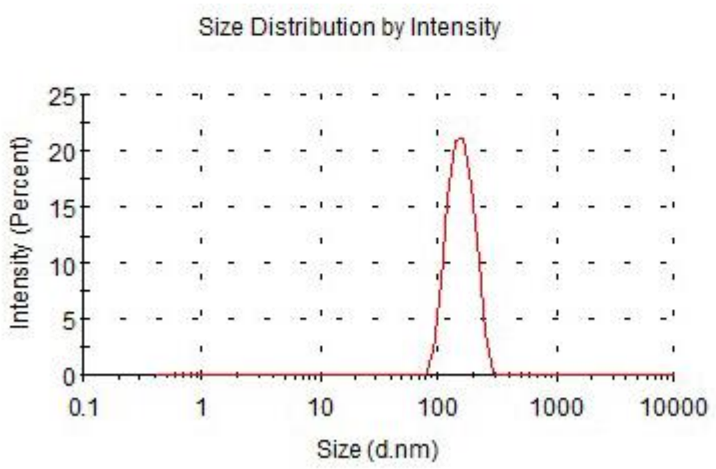

Figure S5. Average particle size of liposome as measured by dynamic light scattering. (Nano ZS90 (ZE N3690), Malvern Instrument Ltd., Malvern, UK).

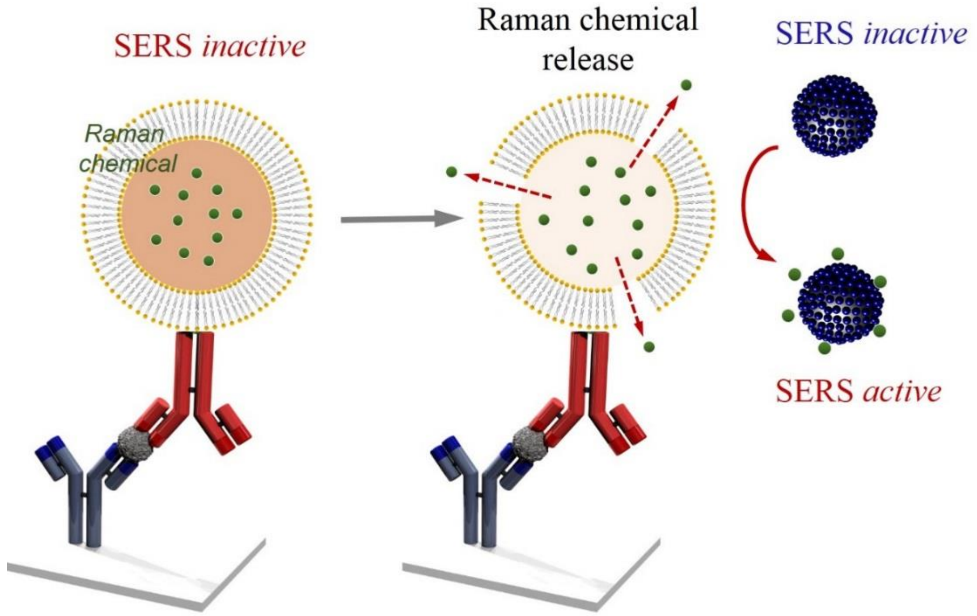

Figure S6. Suggested applications of RLC-encapsulated liposome-enhanced SERS-based immunoassays.
